# Diploid hybrid fish derived from the cross between female Bleeker’s yellow tail and male topmouth culter, two cyprinid fishes belonging to different subfamilies

**DOI:** 10.1186/s12863-019-0781-5

**Published:** 2019-10-23

**Authors:** Shengnan Li, Lihua Xie, Jun Xiao, Liujiao Yuan, Tian Zhou, Kaikun Luo, Chun Zhang, Rurong Zhao, Min Tao, Shaojun Liu

**Affiliations:** 1 State Key Laboratory of Developmental Biology of Freshwater Fish, Hunan Normal University, Changsha, 410081 Hunan China; 2 College of Life Sciences, Hunan Normal University, Changsha, 410081 Hunan China

**Keywords:** Biological characteristics, Diploid hybrid fish, Distant hybridization, Ovary development

## Abstract

**Background:**

Bleeker’s yellow tail (*Xenocypris davidi* Bleeker, YT) and topmouth culter (*Culter alburnus* Basilewsky, TC) are both famous and important economic freshwater fish in China. YT, a kind of omnivorous fish, has strong resistance. TC, a kind of carnivorous fish, has high-quality meat but poor resistance. Distant hybridization can integrate the advantages of both parents. There has been no previous report regarding hybrid fish derived from female YT × male TC. It is expected that hybridization of these two kinds of fish will result in F_1_ hybrids with improved characteristics, such as faster growth rate, stronger resistance, and high-quality meat, which are of great significance in fish genetic breeding.

**Results:**

In this study, we investigated the main biological characteristics of diploid hybrid fish derived from female YT × male TC. The hybrids had an intermediate number of upper lateral line scales between those for YT and TC. The hybrids were diploids with 48 chromosomes and had the same karyotype formula as their parents. The hybrids generated variations in 5S rDNA (designated class IV: 212 bp) and lost specific 5S rDNA derived from the maternal parent (designated class II: 221 bp), which might be related to hybridization. In terms of reproductive traits, all the tested female hybrids exhibited normal gonadal development, and the two-year-old F_1_ females produced mature eggs. However, all the tested testes of the male hybrids could not produce mature sperm. It is possible that the hybrid lineage will be established by back-crossing the fertile female hybrids and their parents.

**Conclusions:**

Obtaining a fertile female hybrid fish made the creation of a new type of fish possible, which was significant in fish genetic breeding.

## Background

In fish, distant hybridization is the mating of two species of fishes that may involve crosses between different species or higher-ranking taxa [1, 2]. Hybridization can combine parental advantages and result in offspring with traits superior to those of the parents, such as faster growth rate, stronger disease resistance, and higher survival rate. Hybridization is universally believed to play a significant role in the formation of polyploidy. For example, previous studies indicated that allotetraploid hybrid lineages (F_3_-F_27_) were obtained from the hybridization of female red crucian carp (*Carassius auratus* red var., RCC) and male common carp (*Cyprinus carpio* L., CC) [1–3]. Autotetraploid fish lineages (F_2_-F_13_) and homologous diploid strains of red crucian carp (F_1_-F_13_) were derived from the hybridization of female RCC and male blunt snout bream (*Megalobrama amblycephala*, BSB) [2, 4]. Allodiploid fish lineages (F_1_-F_5_) were produced from the hybridization of female BSB and male topmouth culter (*Culter alburnus* Basilewsky, TC) [2, 5, 6]. Allodiploid fish lineages (F_1_-F_5_) were also obtained from the hybridization of female TC and male BSB [2, 5, 6].

In the process of speciation, hybridization and genetic introgression between species are a relatively frequent occurrence, particularly in rapidly radiating biological populations, promoting species formation [1, 7–10]. Numerous studies demonstrated that hybrid fish lineages exhibited unique phenotypes and genotypes [1–6]. The formation of distant hybrid lineages is of great importance in genetic breeding and biological evolution.

In eukaryotes, the genes encoding 5S ribosomal RNA (rRNA) are composed of tandem repeat units, and every repeat unit is formed by a highly variable non-transcribed spacer (NTS) region and a highly conserved coding region of the 5S rRNA gene (approximately 120 bp) [11–13]. Many researchers demonstrated that the NTS sequence could be used as a molecular genetic marker for species identification and phylogenetic analysis [14–17]. Hybridization can affect the organization and sequences of the 5S rRNA genes of the hybrid, including base substitutions and insertion-deletions [18]. Moreover, hybridization can induce the loss of the parental-special 5S ribosomal DNA (rDNA) unit and can even form the novel unit. For example, after analysing the 5S rRNA genes in different ploidy-level hybrids derived from female RCC × male TC, He et al. [19] found that a paternal-special unit lost and a novel unit was generated in the hybrid offspring.

In animals, *Sox* genes have a conserved motif encoding a high-mobility group (HMG) DNA-binding domain of 79 amino acids, regulating the binding of specific DNA sequences [20–24]. More than 100 *Sox* genes were found in many organisms, including nematodes, fishes, amphibians, reptiles, birds, mammals and hexapods [24–28]. Through Sox-HMG analysis of 42 types of animals, including birds, reptiles, amphibians, natural fishes, artificial hybrid fishes and hexapods,Chen et al. [27] verified that the HMG domain of the *Sox* gene was specific in various species and was a new and highly conserved molecular marker sequence.

Based on our previous study, we performed inter-subfamily hybridization by crossing female Bleeker’s yellow tail (*Xenocypris davidi* Bleeker, YT) and male TC, possessing the same number of chromosomes (2n = 48). YT and TC are both famous and important economic freshwater fish in China. In zootaxy, YT is an omnivore with strong resistance belonging to the Xenocyprininae subfamily [29]. TC, characterized by its topmouth, is a carnivore with high-quality meat but poor resistance belonging to the Cultrinae subfamily [30, 31]. In terms of shape, YT is round with a long body, and the TC is linear with a long and low body. In this study, diploid hybrids (2nYC) were successfully obtained by mating female YT with male TC, and the new F_1_ hybrids with improved characteristics, such as faster growth rate, stronger resistance, and high-quality meat, might have great significance for fish genetic breeding. This study provided guidance for further study of the general laws of distant hybridization.

## Results

### Fertilization rates and hatching rates

The average fertilization rate and hatching rate of all groups were listed in Table 1. The fertilization rate and hatching rate of the hybrids (YT (♀) × TC (♂)) were 85 and 77%, respectively. The results showed that the fertilization rate and hatching rate of the experimental group (YT (♀) × TC (♂)) was not significantly differently (*P* > 0.05) from those of the control groups (YT (♀) × YT (♂) and TC (♀) × TC (♂)). However, only 2% of the offspring of YT (♀) × TC (♂) survived to adulthood, and only approximately 100 living hybrids were obtained each year.

**Table 1:** Fertilization rate and hatching rate of YT, TC and their hybrid offspring

Groups	Fertilization rate (%)	Hatching rate (%)
YT (♀) × YT (♂)	85.5	70
TC (♀) × TC (♂)	84	69
YT (♀) × TC (♂)	85	77

### Phenotypes of the hybrid and its parents

**Fig. 1:**
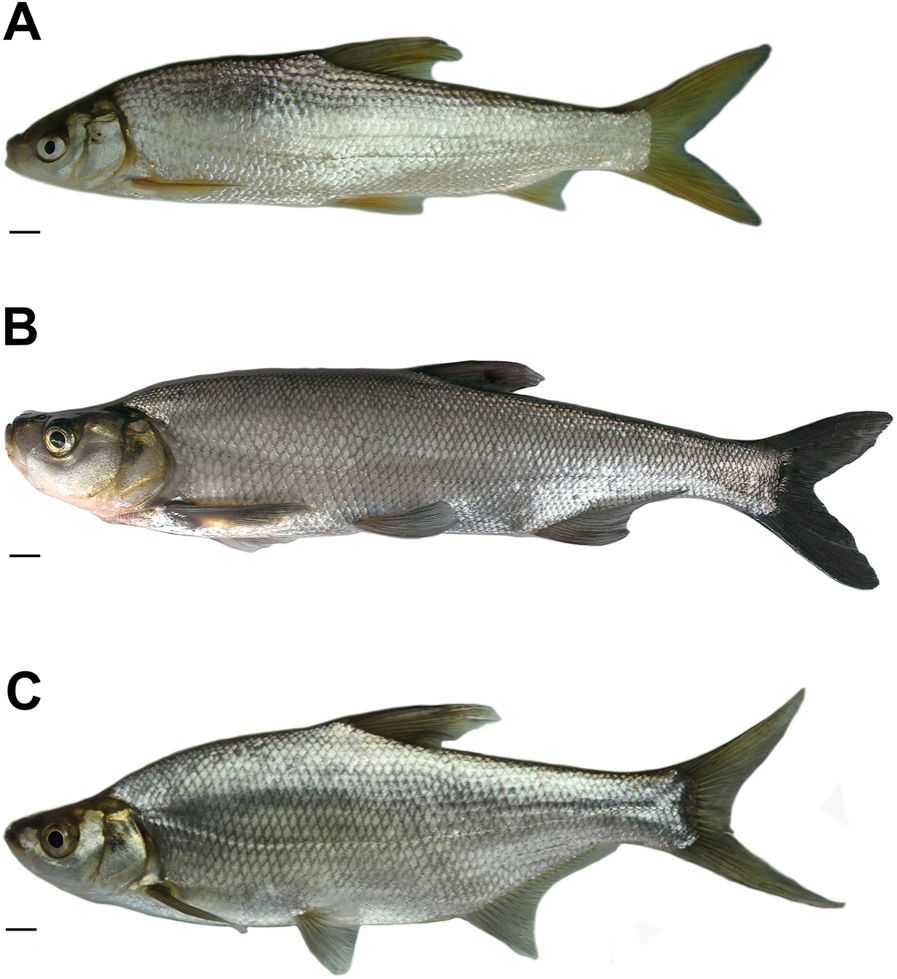
Appearance of YT, TC, and 2nYC. **a** Appearance of YT; **b** Appearance of TC; **c** Appearance of 2nYC. Bar in **a**-**c**, 1 cm

**Fig. 2:**
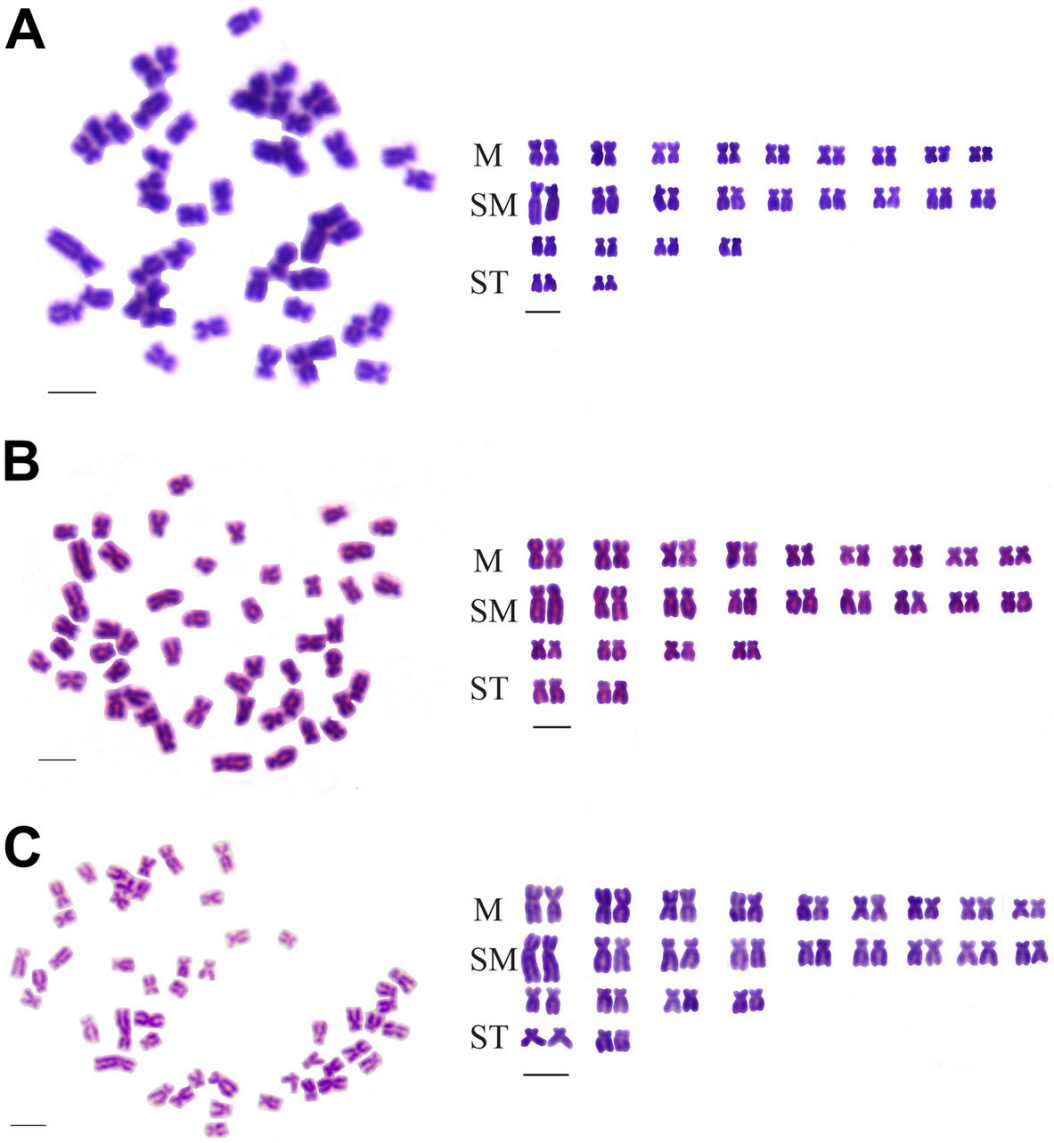
Metaphase chromosome spreads and corresponding karyotypes of YT, TC, and 2nYC. **a** Chromosome number (left) and karyotype (right) of YT; **b** Chromosome number (left) and karyotype (right) of TC; **c** Chromosome number (left) and karyotype (right) of 2nYC. Bar in **a**-**c**, 3 μm

The phenotypes of YT (Fig. 1a), TC (Fig. 1b) and 2nYC (Fig. 1c) were illustrated in Fig. 1, and these results showed that 2nYC was characterized by slight topmouth and high dorsum. The values for the six countable traits (lateral line scales, upper and lower lateral line scales, dorsal fin rays, abdominal fin rays, and anal fin rays) in YT, TC, and 2nYC were presented in Table 2. The number of upper lateral line scales in 2nYC individuals was intermediate (13–14), with values between those for YT (10–12) and TC (16–20), revealing that 2nYC possessed a hybrid-type shape. However, the number of lower lateral line scales in 2nYC was slightly higher than those in the parents, indicating variability in 2nYC.

**Table 2:** Countable traits of YT, TC, and 2nYC

Fish type	Number of lateral line scales	Number of upper lateral line scales	Number of lower lateral line scales	Number of dorsal fin rays	Number of abdominal fin rays	Number of anal fin rays
YT	60–67	10–12	6–8	III + 7–9	8–9	III + 11–13
TC	80–92	16–20	6–7	III + 7	9	III + 20–23
2nYC	64–66	13–14	9–10	III + 8	9–10	III + 22–23

The average BL/WL, HL/BL, BH/BL, HH/HL, HH/BH, and CPH/CPL ratios were shown in Table 3. We discovered significant differences (*P* < 0.01) between YT and 2nYC for all measurable traits, indicating that the appearance of 2nYC was strikingly different from that of YT. Likewise, there were significantly differences (*P* < 0.01) in the BL/WL, HL/BL, HH/HL, HH/BH, and CPH/CPL ratios between TC and 2nYC, suggesting that 2nYC was different from TC in terms of most phenotypic traits. The ratios of HH/HL and HH/BH for 2nYC were numerically intermediate between those for YT and TC, indicating that 2nYC exhibited a variable hybrid-type shape for these morphological traits. Peculiarly, the HL/BL ratio was significantly higher in 2nYC than in either YT or TC, and the CPH/CPL ratio in 2nYC was lower than that in either YT or TC.

**Table 3:** Measurable traits of YT, TC, and 2nYC

Fish type	BL/WL^a^	HL/BL^a^	BH/BL^a^	HH/HL^a^	HH/BH^a^	CPH/CPL^a^
YT	0.82 ± 0.02	0.20 ± 0.13	0.27 ± 0.15	0.79 ± 0.04	0.57 ± 0.08	0.65 ± 0.13
TC ^b^	0.84 ± 0.02	0.20 ± 0.22	0.25 ± 0.13	0.46 ± 0.29	0.44 ± 0.26	0.85 ± 0.11
2nYC	0.83 ± 0.01	0.22 ± 0.15	0.25 ± 0.44	0.61 ± 0.08	0.52 ± 0.09	0.53 ± 0.17

^a^*BL* body length, *WL* whole length, *HL* head length, *BH* body height, *HH* head height, *CPH* caudal peduncle height, *CPL* caudal peduncle length

^b^20 samples were counted for each kind of fish

### DNA content

We used the DNA content of the parents (YT and TC) as the control. The comparison of the DNA content of YT, TC, and 2nYC was presented in Table 4. The results showed that the DNA content of 10 2nYC from YT (♀) × TC (♂) was equal (*P* > 0.05) to the sum of half YT and half TC, suggesting that 2nYC, which might receive half of their chromosomes from YT and half the TC, were diploid, similar to the parent fish.

**Table 4:** Mean DNA content of YT, TC, and 2nYC

Fish type	Mean DNA content^a^	Deviation	Variation coefficient (%)	Ratio	
				Observed ratio	Expected ratio
YT1	66.71	0.072	3.37	2nYC1/(0.5 YT1 + 0.5 TC1) = 1.04^b^	1
YT2	65.29	−1.348	5.12		
YT3	67.64	1.002	4.19		
YT4	65.43	−1.208	4.80		
YT5	68.12	1.482	3.12		
TC1	65.07	0.926	6.76		
TC2	64.10	−0.044	4.56		
TC3	62.73	−1.414	5.65		
TC4	64.53	0.386	4.15		
TC5	64.29	0.146	3.84		
2nYC1	68.40	−0.517	6.21		
2nYC2	70.18	1.263	4.32		
2nYC3	71.42	2.503	7.53		
2nYC4	67.19	−1.727	4.64		
2nYC5	69.98	1.063	2.13		
2nYC6	69.18	0.263	3.56		
2nYC7	66.23	−2.687	4.11		
2nYC8	67.87	−1.047	5.98		
2nYC9	70.59	1.673	7.87		
2nYC10	68.13	−0.787	6.95		

^a^The intensity of fluorescence (unit, channel)

^b^The observed ratio was not significantly different (*P* > 0.05) from the expected ratio

### Chromosome number of the hybrid and its parents

The chromosome number distribution of YT, TC, and 2nYC was shown in Table 3. Of the YT individuals examined, 98% of the chromosomal metaphase spreads had 48 chromosomes, indicating that they were diploids with 48 chromosomes (2n = 48) (Table 5, Fig. 2a). Of the TC individuals examined, 93% of the chromosomal metaphase spreads possessed 48 chromosomes, showing that they were diploids with 48 chromosomes (2n = 48) (Table 5, Fig. 2b). Additionally, 95.5% of the chromosomal metaphase spreads in 2nYC possessed 48 chromosomes, indicating that they were also diploids with 48 chromosomes (2n = 48) (Table 5, Fig. 2c). In addition, according to the classification standards reported by Levan et al. [32], YT, TC, and 2nYC possessed the same karyotype formula of 18 m + 26sm + 4st, including a pair of large submetacentric chromosomes (Fig. 2).

**Table 5:** Distribution of chromosome number in YT, TC, and 2nYC

Fish type	Number of fish	Number of metaphase spreads	Distribution of chromosome number	
			< 48	48
YT	5	100	2	98
TC	5	100	7	93
2nYC	10	200	9	191

### Molecular analysis of polymorphisms in 5S rDNA sequences

**Fig. 3:**
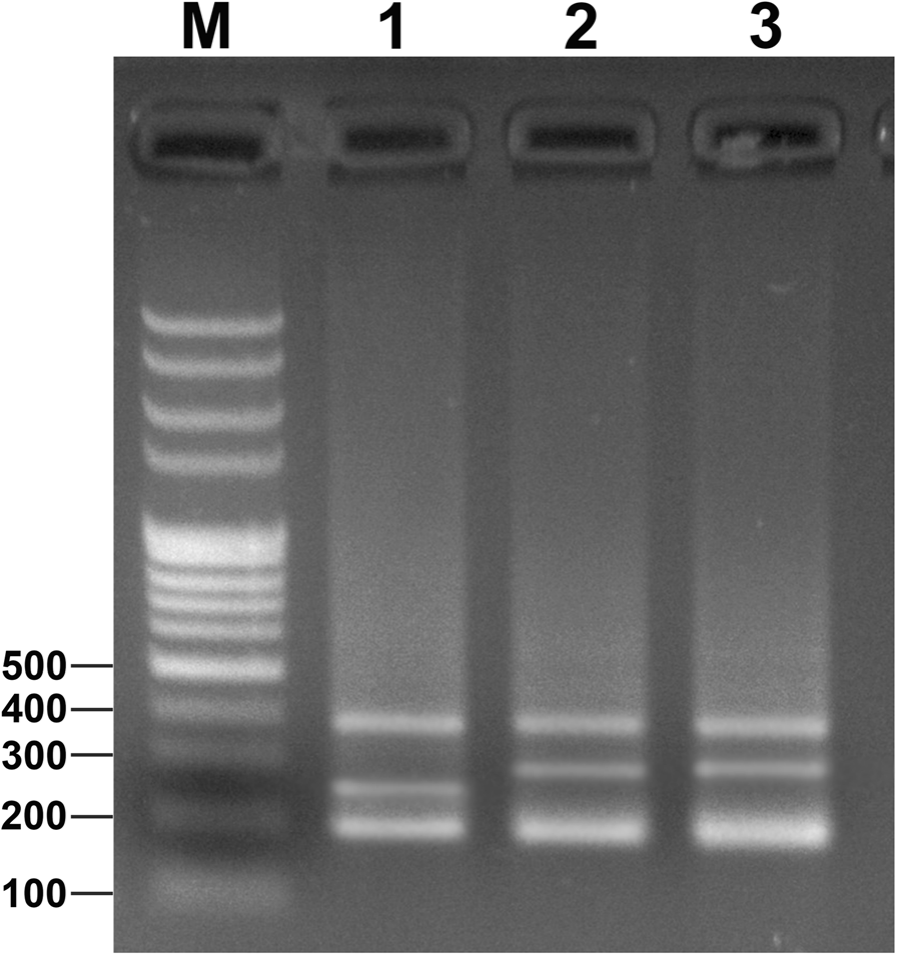
PCR-generated 5S rDNA products obtained from YT, TC, and 2nYC. M, DNA ladder markers (100 bp increments); lane 1, three bands (approximately 200, 250, and 400 bp) from YT; lane 2, three bands (approximately 200, 280, and 400 bp) from TC; lane 3, three bands (approximately 200, 280, and 400 bp) from 2nYC

The primers (5SP1 and 5SP2R) were used to amplify DNA fragments from YT, TC, and 2nYC, and the results of agarose gel electrophoresis showed that YT had three bands (approximately 200, 250, and 400 bp), TC had three bands (approximately 200, 280, and 400 bp), and 2nYC had three bands (approximately 200, 280, and 400 bp) (Fig. 3). A total of 120 clones (30 from YT, 30 from TC and 60 from 2nYC) were generated to further evaluate the differences in 5S rDNA patterns (Table 6). The sequencing results indicated that YT had three different sizes (188, 221, and 376 bp), TC had three different sizes (188, 286, and 376 bp), and 2nYC had four different sizes (188, 212, 287 and 376 bp) (Table 6). Sequence analysis revealed that 2nYC had two similarly sized 5S rDNA sequences (188 and 212 bp) that were indistinguishable on the agarose gel, where they appeared as a single band of approximately 200 bp.

**Table 6:** Results of 5S sequencing

Species	Total number of sequenced clones	PCR fragments			
		~ 200 bp	~ 250 bp	~ 280 bp	~ 400 bp
YT	30	Ten sequenced clones of 188 bp	Ten sequenced clones of 221 bp	Absent	Ten sequenced clones of 376 bp
TC	30	Ten sequenced clones of 188 bp	Absent	Ten sequenced clones of 286 bp	Ten sequenced clones of 376 bp
2nYC	60	Nine sequenced clones of 188 bp; eleven sequenced clones of 212 bp	Absent	Twenty sequenced clones of 287 bp	Twenty sequenced clones of 376 bp

**Fig. 4:**
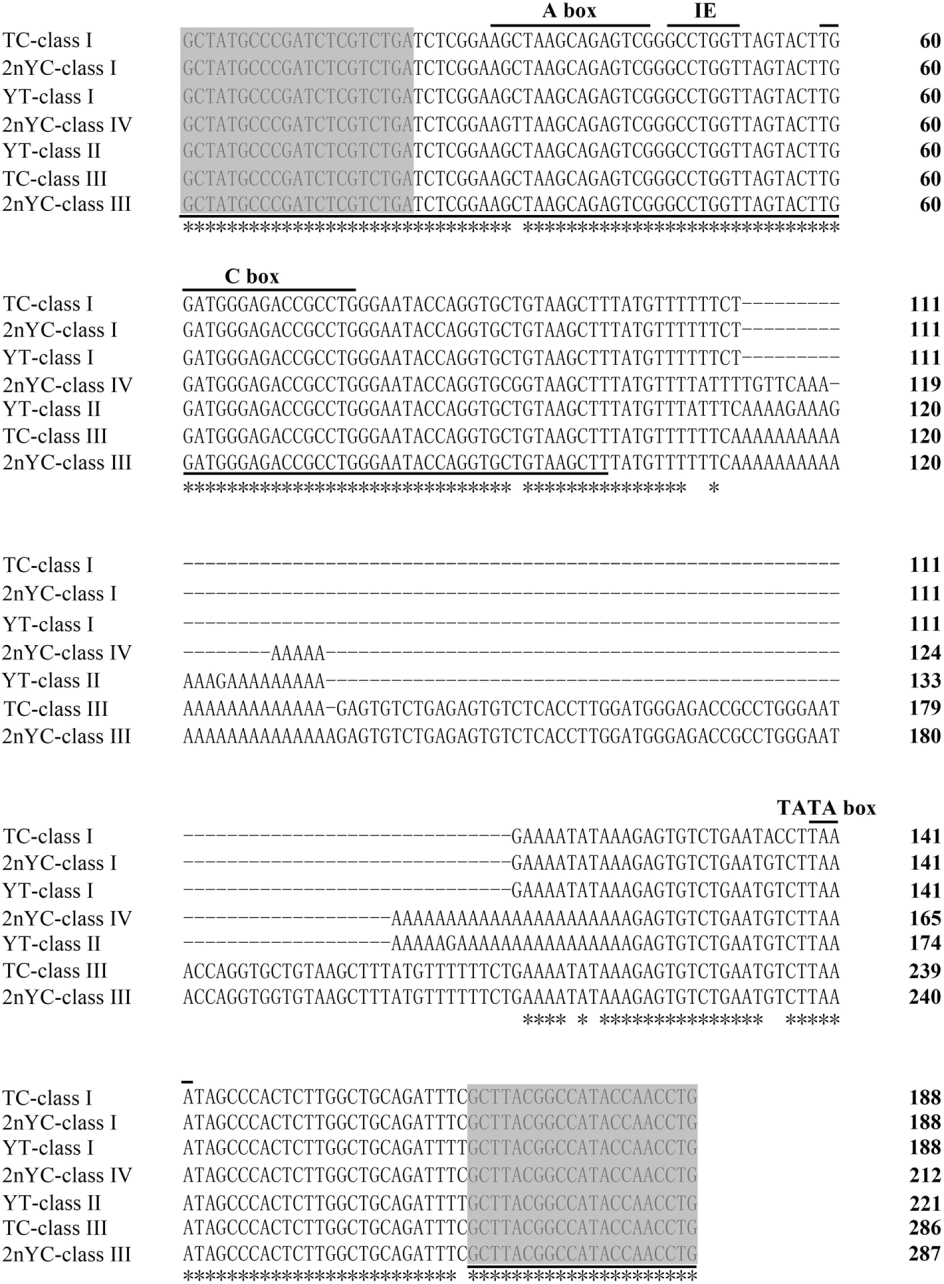
Comparison of class I, class II, class III, and class IV from YT, TC, and 2nYC. The coding region of the 5S rDNA is underlined, and the primers used to amplify 5S rDNA are shaded. Asterisks indicate consistent nucleotides

BLASTn was used for sequence analysis, and all fragments of YT, TC, and 2nYC were shown to be 5S rDNA repeat units that consisted of a highly conserved coding region (120 bp) and highly variable NTS regions of different lengths. In YT, the two monomeric 5S rDNA classes (designated class I: 188 bp and class II: 221 bp) were characterized by diverse NTS types (designated NTS-I: 68 bp and NTS-II: 101 bp). In TC, the two monomeric 5S rDNA classes (designated class I: 188 bp and class III: 286 bp) were characterized by disparate NTS types (designated NTS-I: 68 bp and NTS-III: 166 bp). 2nYC inherited class I (188 bp) and class III (287 bp) from its parents but derived a new 5S rDNA class (designated class IV: 212 bp) with a novel NTS type (designated NTS-IV: 92 bp). However, no maternal-specific 5S rDNA sequence (class II) was found in 2nYC. All 5S rDNA sequences were submitted to GenBank, and their accession numbers were listed in Table 7. A comparison of class I from YT and 2nYC showed a base substitution, and two base substitutions were found between TC and 2nYC (Fig. 4). A comparison of class III from TC and 2nYC revealed a base substitution (C-G) in the NTS regions and a deletion-insertion of an adenine residue (Fig. 4). A comparison of class II from YT and class IV from 2nYC showed two base substitutions (C-T and T-G) in the coding regions, even in the A box, but not in the internal element (IE) or C box. Moreover, there were significant differences in the non-coding regions, mainly showing an insertion-deletion and obvious nucleotide variations (Fig. 4). All the internal control regions that function as gene promoters (A box, IE and C box) were detected. Within all the NTS sequences of YT, TC, and 2nYC, the TATA box control element was detected and had been modified to TAAA. The poly-T tract required for transcription termination was also detected (Fig. 4).

**Table 7:** GenBank accessions of the 5S rDNA sequences from YT, TC, and 2nYC

DNA fragments (bp)	GenBank accession number of the sequences		
	YT	TC	2nYC
188	MN158655	MN158657	MN158659
212, 221	MN158656	Absent	MN158660
286, 287	Absent	MN158658	MN158661

### DNA fragments of *Sox* genes

**Fig. 5:**
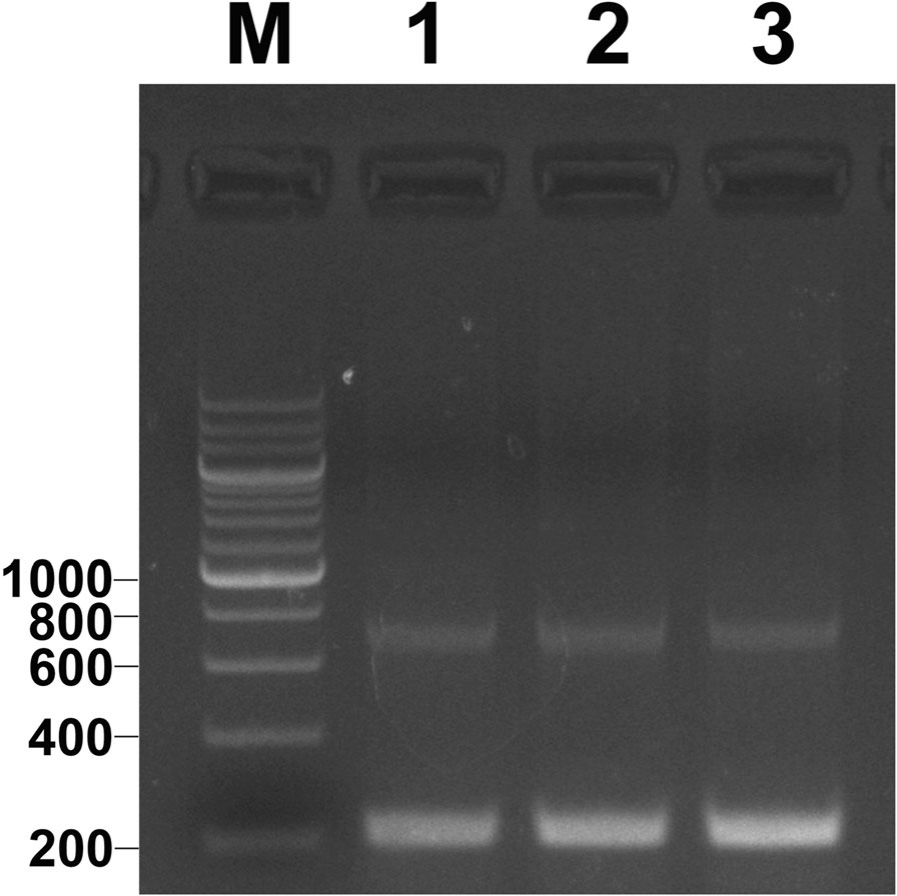
PCR-generated *Sox* products obtained from YT, TC, and 2nYC. M, DNA ladder markers (200 bp increments); lane 1, two bands (approximately 200 and 700 bp) from YT; lane 2, two bands (approximately 200 and 700 bp) from TC; lane 3, two bands (approximately 200 and 700 bp) from 2nYC

Primers (HMG (+) and HMG (−)) were used to amplify DNA fragments from YT, TC, and 2nYC, and the results of agarose gel electrophoresis showed that there were two bands (approximately 200 and 700 bp) in YT, TC, and 2nYC (Fig. 5). Twenty clones were selected for sequencing and analysis of each band to further assess the differences in *Sox* patterns, and there were three different DNA fragments in YT (215, 215, and 710 bp), three in TC (215, 215, and 726 bp), and four in 2nYC (215, 215, 710, and 726 bp).

**Fig. 6:**
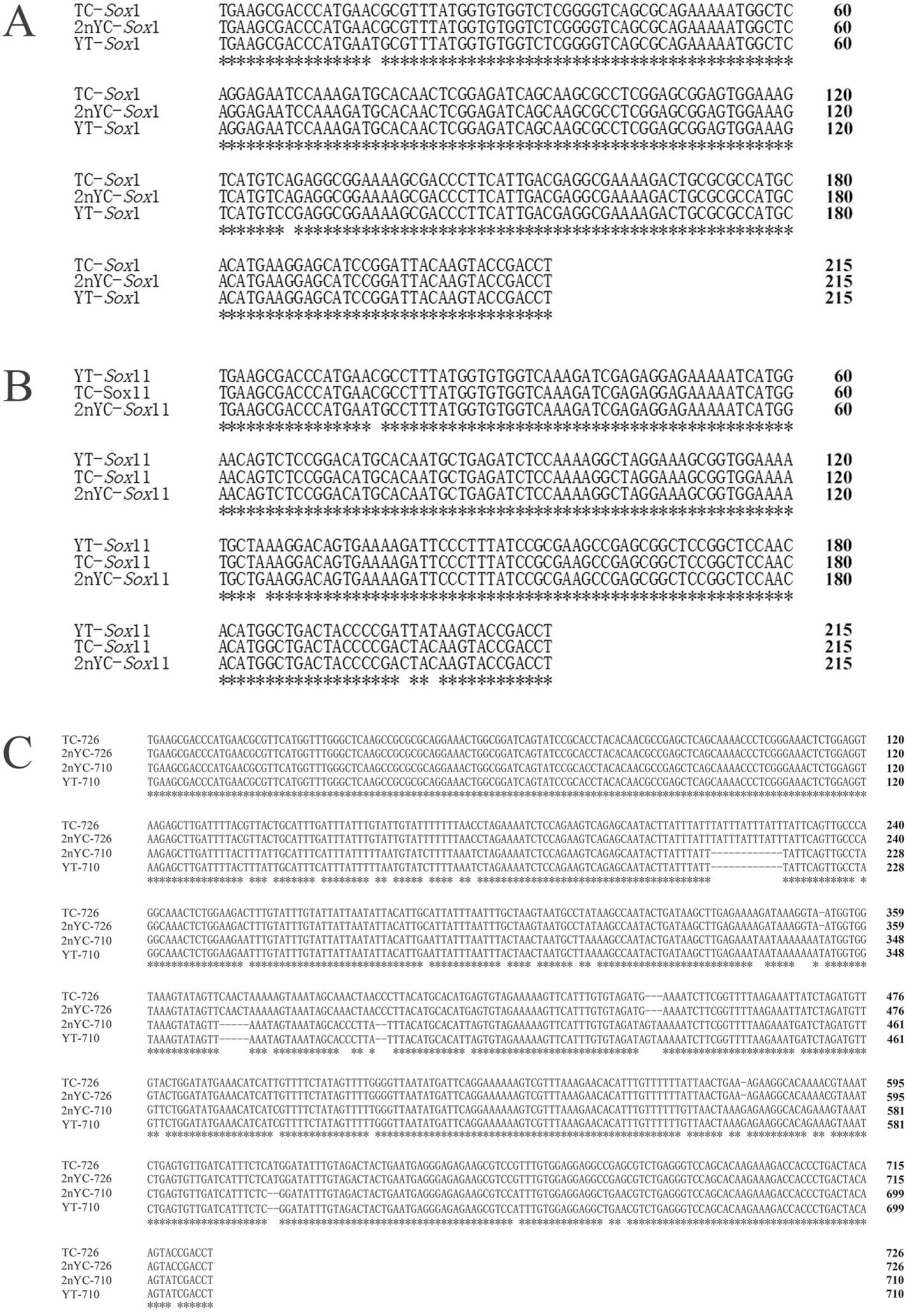
Alignment of *Sox* sequences. **a** Alignment of *Sox*1 sequences from YT, TC, and 2nYC; **b** Alignment of *Sox*11 sequences from YT, TC, and 2nYC. **c** Alignment of *Sox*9 sequences from YT, TC, and 2nYC. Asterisks indicate consistent nucleotides

BLASTn was employed for sequence analysis. The resulting data were summarized in Table 8 and indicated that there were two different DNA fragments with lengths of 215 bp in 2nYC and its parents, one belonging to the *Sox*1 gene and the other belonging to the *Sox*11 gene. A 710 bp DNA fragment existed in YT and 2nYC and belonged to the *Sox*9 gene, whereas a 726 bp DNA fragment in TC and 2nYC also belonged to the *Sox*9 gene. We compared all fragments belonging to *Sox* genes in YT, TC, and 2nYC. In the *Sox*1 sequences, which consisted of a 215 bp DNA fragment, two base substitutions were found between YT and TC; the sequence in 2nYC was exactly the same as that in the paternal TC and had two base substitutions compared with that in the maternal YT (Fig. 6a). In the *Sox*11 sequences, which consisted of a 215 bp DNA fragment, two base substitutions were found between YT and TC; similarly, the sequence in 2nYC had two base substitutions compared with that in the parental TC and four base substitutions compared with that in the maternal YT (Fig. 6b). In addition, the 710 bp gene fragment in 2nYC, which was derived from the maternal YT, exhibited 100% similarity with the 710 bp gene fragment in YT. The 726 bp gene fragment in 2nYC, which was derived from the paternal TC, exhibited 100% similarity with the 710 bp gene fragment in TC (Fig. 6c).

**Table 8:** *Sox* genotypes of 2nYC and its parents

DNA fragments	YT	TC	2nYC
215 bp	*Sox*1	*Sox*1	*Sox*1
215 bp	*Sox*11	*Sox*11	*Sox*11
710 bp	*Sox*9	Absent	*Sox*9
726 bp	Absent	*Sox*9	*Sox*9

### Chromosomal loci of 5S rDNA

**Fig. 7:**
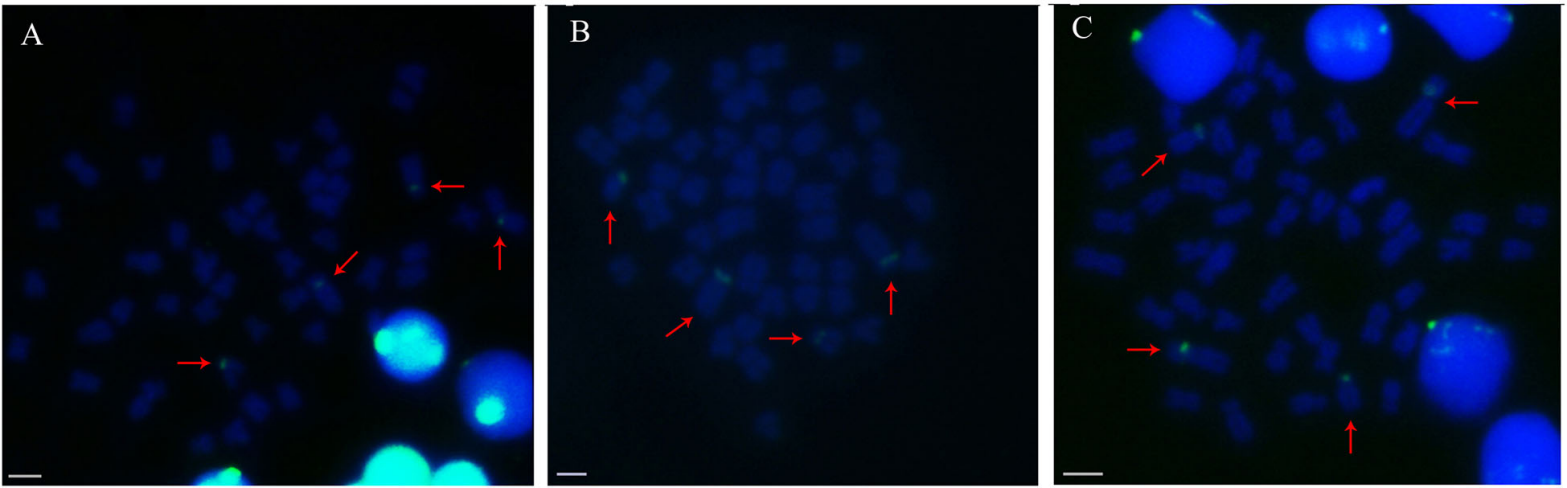
FISH hybridization signals in YT, TC, and 2nYC with 188 bp 5S rDNA as a probe. Fluorescein isothiocyanate (FITC)-conjugated avidin was used to detect signals, and 4′,6-diamidino-2-phenylindole (DAPI) was used to stain all the metaphase chromosomes. The red arrows manifest the hybridizing signals. The four hybridizing signals in YT **a**, TC **b**, and 2nYC **c** are shown. Bars in **a**-**c**, 3 μm

The 188 bp 5S rDNA unit was used as a hybridization probe with the mitotic metaphase chromosomes of YT, TC, and 2nYC. In the present investigation using FISH, four fluorescent signals of 5S rDNA were found in most of the metaphase spreads of YT, TC and their hybrid offspring. Comparison of the chromosomal location indicated that two signals were positioned on a pair of the largest submetacentric chromosomes, and the other two were on a pair of subtelocentric chromosomes (Fig. 7). All the 5S rDNA loci were near the centromere. These results indicated that the 188 bp 5S rDNA unit could be used as an effective marker in future investigations of the ploidy of a hybrid resulting from YT and TC.

### Analysis of hybrid gonadal development

**Fig. 8:**
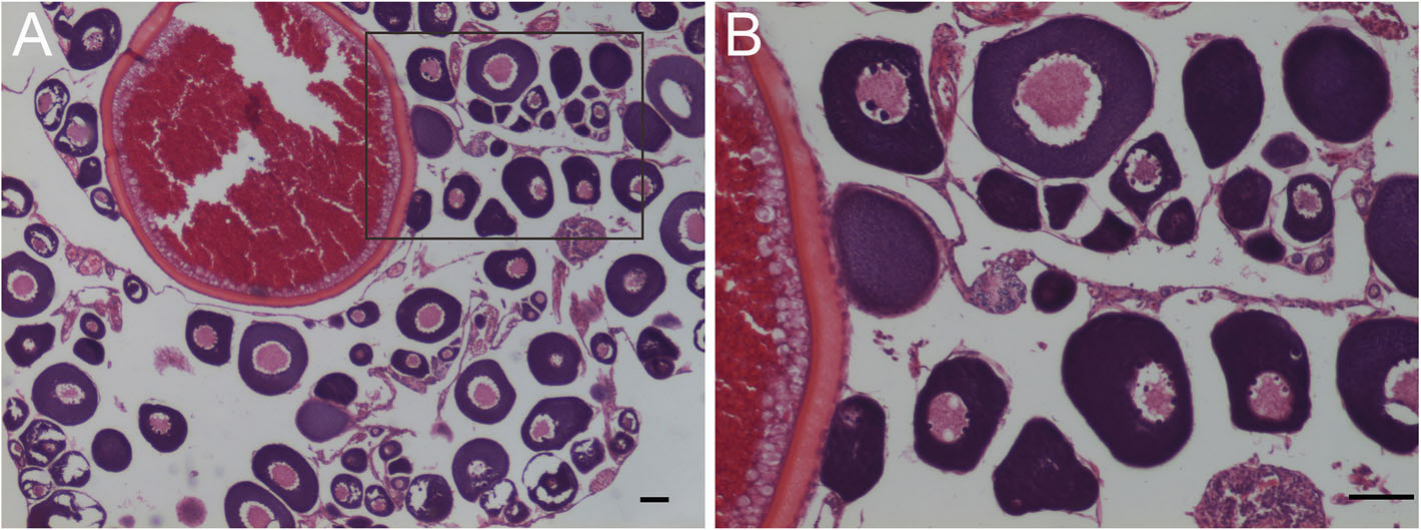
Ovary microstructure of 2nYC. **a** Histological section of the ovary of 2nYC. The part in the black frame is enlarged in **b**. Bars in **a** and **b**, 50 μm

Figure 8 presented the ovarian microstructure of 2-year-old 2nYC. At the time of analysis, the ovaries had developed normally and had entered phase IV. Meanwhile, a large number of oocytes at phase II were observed, and a small number of oogonia were found. However, all the tested testes of the male hybrids could not produce mature sperm.

## Discussion

Distant hybridization, i.e., the translation of the haploid genome of one species into another species, may lead to genotypic and phenotypic changes in the offspring. Moreover, distant hybridization is proven to be a useful way to create new fish species in fish genetic breeding, fish reproduction, and aquaculture. Due to nuclear-cytoplasmic incompatibility and other reasons, it is difficult to obtain fish offspring via distant hybridization between orders, families, and subfamilies. The obtained hybrid offspring were usually considered to be the result of gynogenesis. However, studies on the fertilization cytology of *Ctenopharyngodon idellus* (grass carp, GC) (♀) × *Megalobrama terminalis* (♂) [33] and CC (♀) × GC (♂) [34] showed that there were also normal fertilization events that occurred between these distantly related species. Previous studies indicated that diploid hybrid offspring were sparse in the F_1_ generation of some distant crosses [1, 6, 33–37]. For example, no diploid hybrid offspring were obtained among F_1_ hybrids of RCC (♀) × BSB (♂) [1, 35], RCC (♀) × YT (♂) [1, 6], etc. Moreover, even if diploid hybrid offspring were produced among F_1_ hybrids of female RCC and male TC, their fertility was not good enough [6, 19]. In the present study, we obtained diploid F_1_ hybrid offspring by mating of female YT with male TC, which belonged to different subfamilies but with the same chromosome number. The ploidy level of 2nYC was verified by measuring the DNA content, counting the chromosomal number (Fig. 2c), observing the karyotype (Fig. 2c), and FISH analysis (Fig. 7c). All of the results above confirmed that 2nYC was diploid with 48 chromosomes. Histological observation showed that 2nYC underwent normal ovarian development (Fig. 8) and produced mature eggs. Similarly, previous studies indicated that fertile diploid hybrid offspring were obtained by mating of female BSB and TC, and their parents possessed the same chromosome number [38]. Therefore, we speculated that the formation of fertile diploid hybrid offspring was related to the chromosome number of the parents. Parents with the same chromosome number is a prerequisite for the generation of diploid hybrid offspring. In addition, we could obtain triploid hybrids in the F_1_ generation of hybridization when the number of maternal chromosomes was equal to that of paternal chromosomes, as described in previous studies [31, 38, 39]. However, there was no triploid generated by the hybridization of female YT and male TC in this study. We speculated that the absence of triploid formation might be attributed to the reason that the second meiosis could be normally carried out and the second polar body could be normally emitted.

In the process of distant hybridization, the F_1_ hybrid offspring tended to integrate favourable parental traits and exhibit heterosis [40, 41]. In terms of countable traits, the 2nYC hybrids presented an intermediate number of upper lateral line scales compared to YT and TC (Table 2). In terms of measurable traits, the 2nYC hybrids exhibited a lower CPH/CPL ratio compared to their parents (Table 3). These traits could act as morphological markers to distinguish the hybrids from their parents. In short, the hybrids showed hybrid characteristics and hybrid heterosis. Moreover, our results provided support for the hybrid origin of the offspring of YT (♀) × TC (♂), rather than the outcome of gynogenesis or androgenesis. Moreover, 2nYC exhibited the high fertilization rate and the hatching rate but the low adult survival rate.

Compared with the 5S rDNA results of He et al. [19] and Wu et al. [42], our results showed that different TC individuals exhibited genetic diversity. Other studies also confirmed that TC exhibited some genetic variability [43]. Previous studies showed that hybridization could lead to variation in the structure and sequence of 5S rDNA, including base substitutions and deletion-insertions in the NTS sequence, parental genome-specific loss, and formation of new 5S rDNA classes [19]. In this study, two types of 5S rDNA classes were detected in YT and TC, which favoured the hypothesis that the presence of two types of 5S rDNA classes was a general trend in 5S rRNA gene organization in the fish genome [44]. However, 2nYC had three types of 5S rDNA classes, partially inheriting 5S rDNA classes from their parents. 2nYC inherited the 188 bp and 287 bp fragments from its parents but also obtained a novel 5S rDNA unit (212 bp), which revealed heredity and variability in 2nYC at the molecular level and that these variations were related to hybridization. To reduce incompatible parental genetic material, rapid genetic recombination was needed in 2nYC hybrids, which led to the presence of a novel 5S rDNA and the absence of parental-specific 5S rDNA. Hybridization can lead to genomic changes, including changes in gene expression, chromosome structure and genome size [45]. In the present study, we observed two nucleotide mutations in the coding region of the hybrid offspring, even in the A box (Fig. 4). We also detected nucleotide polymorphisms, including base insertion-deletions and substitutions in the NTS sequences of the hybrid offspring (Fig. 4). Numerous studies indicated that the NTS sequence could be used as a molecular genetic marker for species identification and phylogenetic analysis [14–18]. Comparative analysis of the NTS sequence demonstrated that 2nYC hybrids had three types of NTS sequences (NTS-I, NTS-III, and NTS-IV), among which NTS-IV showed an insertion-deletion and obvious nucleotide variations compared to NTS-II. We speculated that this phenomenon might be the result of the allele being inherited from the female parent and the production of recombinant mutations. Meanwhile, NTS-I of 2nYC was highly homologous to those of YT and TC, and NTS-III of 2nYC was also highly homologous to that of TC. These results indicated that distant hybridization could induce base insertions, deletions and substitutions in the NTS sequence and that these variations in the NTS sequence were always species-specific. Thus, we determined that the NTS sequence could act as an effective molecular marker to distinguish hybrid offspring and their parents. Similarly, 2nYC inherited *Sox*9 (710 bp) from YT and *Sox*9 (726 bp) from TC, which suggested the heredity in 2nYC. Thus, the *Sox* gene fragments could also serve as feasible molecular genetic markers to distinguish hybrids and their parents.

FISH is a valid method for determining the chromosomal location of 5S rDNA [46]. This technology, combined with cytogenetic and molecular genetic methodologies, was proven to be very useful for establishing cytogenetic maps and performing genomic studies [47, 48]. In fish, 5S rDNA can be found in more than one single chromosome pair [49]. A total of 41 species of representative cichlids were mapped to 5S rRNA genes to obtain an improved understanding of the genomic organization of rRNA genes and chromosomal evolution [50]. In the genus *Leporinus*, different 5S rDNA arrays characterized two possible 5S rRNA gene subfamilies that evolved independently in the genome [51]. In this study, the chromosomal localization of the 5S rRNA gene unit revealed that there were two signals in a haploid chromosome set, one in the largest submetacentric chromosome and the other in the subtelocentric chromosome. Therefore, the 188 bp 5S rDNA probe could be used to detect the ploidy level of the hybrids, which were derived from YT or TC.

## Conclusions

This research offered solid genetic evidence to support the successful formation of diploid hybrids, and 2nYC exhibited heredity and variability, as determined by analysis of morphological traits, ploidy, 5S rDNA, *Sox*, and fertility. The production of fertile female hybrid fish not only provided the possibility of creating a new type of fish but also expanded the range of genetic material available for breeding. In the future, we will generate a hybrid lineage by back-crossing the female diploid hybrids with their parents. In addition, these results further revealed the influence of hybridization on the organization and genetic variation in 5S rDNA multigene families.

## Methods

### Animals and crosses

Sexually mature YT and TC were obtained from the Engineering Research Center of Polyploid Fish Breeding and Reproduction of the State Education Ministry at Hunan Normal University. All experiments performed were approved by the Animal Care Committee of Hunan Normal University. Approval was acquired for the Administration of Affairs Concerning Animal Experimentation guidelines from the Science and Technology Bureau of China. To minimize suffering, 100 mg/L MS-222 (Sigma-Aldrich, St Louis, MO, USA) was used to anaesthetize fish before dissection.

During the reproductive seasons since 2010, crosses between female YT and male TC were performed. The mature eggs were fertilized with the mature sperm, and the embryos were incubated in freshwater at 22–26 °C. 2nYC (F_1_) was obtained in this cross-group. Meanwhile, self-mating of both YT and TC was conducted as a control. Concrete implementation was performed based on the patented “A method of distant crossing between *Xenocypris davidi* and *Erythroculter ilishaeformis*” (patent no. ZL 2012 1 0449551.7) [52]. Two thousand embryos were selected at random from each group to examine the fertilization rate (number of embryos at the gastrula stage/2000 × 100%) and hatching rate (number of hatched fry/2000 × 100%). The data from six parallel experiments for each group were analysed by analysis of variance (ANOVA) using SPSS Statistics 19.0 (IBM, USA). The hatched fry was transferred to a pond for further cultivation.

### Measurement of morphological traits

At 2 years of age, 20 YT, 20 TC, and 20 2nYC individuals were taken at random for morphological examination. The examination standards followed a kind of schematic diagram of the studied seven measurable traits [53]. Six countable traits of each fish (lateral line scales, upper and lower lateral line scales, dorsal fin rays, abdominal fin rays, and anal fin rays) were recorded. Seven measurable traits (whole length, body length and height, head length and height, and caudal peduncle length and height) were also recorded (accurate to 0.1 cm). The average ratios of body length to whole length (BL/WL), head length to body length (HL/BL), body height to body length (BH/BL), head height to head length (HH/HL), head height to body height (HH/BH), and caudal peduncle height to caudal peduncle length (CPH/CPL) were calculated. SPSS Statistics 19.0 (IBM, USA) was used to conduct ANOVA and pairwise comparisons of the data between every two kinds of fish in YT, TC, and 2nYC.

### Ploidy determination of the hybrid

Peripheral blood was collected from the caudal veins of 5 YT, 5 TC and 10 2nYC individuals with the age of more than six months from each group using a syringe containing sodium heparin and then treated according to the method described by Liu et al. [35]. Blood samples were subjected to nuclear extraction and 4′,6-diamidino-2-phenylindole (DAPI) DNA staining with CyStain DNA 1 Step (Partec, Germany) and then filtered with a nylon filter (Partec, Germany). A flow cytometer (Cell Counter Analyzer, Partec, Germany) was used to measure the mean comparative DNA content, and RCC was used as the control. The mean DNA content of each sample was measured under the same conditions. Eventually, a *χ*^2^ test using SPSS Statistics 19.0 software (IBM, USA) with Yate’s correction was used to examine the deviation in the ratios of the mean DNA content of 2nYC to the sum of that from YT and TC from expected ratio values.

### Preparation of chromosome spreads

Chromosome preparation was performed with cultured peripheral blood cells of the hybrids and their parents as described by Huang et al. [54] with minor modifications. For each fish sample, 100, 100, and 200 metaphase spreads (20 spreads per sample) from YT, TC, and 2nYC were examined, respectively. A total of 0.2 ml of blood was collected using a syringe and cultured in nutrient solution (83% RPMI 1640, 15% foetal bovine serum, 0.002% heparin sodium, phytohemagglutinin, 0.5% penicillin and 10,000 U/ml streptomycin) at 27 °C with 5% CO_2_ for 72 h, adding colchicine (final concentration, 0.6 μg/ml) for 3.5 h before the end of cultivation. Cells were harvested by centrifugation, and the erythrocytes were ruptured by hypotonic treatment with 0.075 M KCl at 37 °C for 30–40 min and then fixed in 3:1 methanol-acetic acid with three changes. Cells were dropped onto a cold and wet slide, air-dried and stained with 4% Giemsa solution for 30 min. All the images were obtained using an oil lens at a magnification of 330×. High-quality metaphase spreads were photographed for karyotype analysis. The lengths of the entire chromosomes, as well as the long and short arms, were measured. Chromosomes were classified according to Levan et al. [32].

### Cloning of the 5S rRNA genes and sox genes

Total genomic DNA of YT, TC, and 2nYC was isolated from the whole blood collected from the fish caudal vein based on the method described by Sambrook et al. [55]. A pair of primers (5SP1, 5′-GCTATGCCCGATCTCGTCTGA-3′; 5SP2R, 5′-CAGGTTGGTATGGCCGTAAGC-3′) was designed as described by Qin et al. [18] and synthesized by Sangon (Shanghai, China), and the 5S rRNA genes were directly amplified from the genomic DNA according to a previous study [19]. Similarly, a set of degenerate primers (HMG (+), 5′-TGAAGCGACCCATGAA(C/T) G-3′; HMG (−), 5′-AGGTCG(A/G)TACTT(A/G)TA(A/G)T-3′) was designed by Chen et al. [26] and synthesized by Sangon (Shanghai, China), and polymerase chain reaction (PCR) amplification of the HMG-box DNA fragments of the *Sox* genes was performed based on the method described by Chen et al. [26]. The PCR amplification products were separated on a 1.5% agarose gel using TBE buffer, purified using a gel extraction kit (UNIQ-10 Column DNA Gel Extraction Kit, Sangon), and cloned into the pMD18-T vector (TaKaRa, Dalian, China). The recombinant plasmids with the DNA fragments were transferred into competent *Escherichia coli* DH5α, and positive clones were sequenced by Sangon. For the purpose of analysing sequence homology and variation among the amplified products from YT, TC, and 2nYC, Bioedit [56] and Clustal W2 software [57] were used to align sequences.

### Fluorescence in situ hybridization

Fluorescence in situ hybridization (FISH) probes for the 5S rRNA gene were constructed for YT. The PCR and temperature adjustments were executed as previously described by Qin et al. [46] with minor modifications. The temperature profile was as follows: initial denaturation step at 94 °C for 5 min, followed by 30 cycles of 94 °C for 30 s, 60 °C for 30 s, and 72 °C for 1 min, with a final extension step at 72 °C for 10 min. The purified PCR products were labelled by Dig-11-dUTP (using the Nick Translation Kit, Roche, Germany) to produce FISH probes, and FISH was carried out as described by He et al. [19]. After treatment with 30 μg/ml RNase A in 2 × SSC for 30 min at 37 °C, the slides with chromosome metaphase spreads were denatured in 70% deionized formamide/2 × SSC for 2 min at 70 °C, dehydrated in 70, 90 and 100% ethanol for 5 min each, and then air-dried. The hybridization mixture (approximately 100 ng of labelled probes, 50% formamide, 10 mg dextran sulfate/ml and 2 × SSC) was denatured in boiling water for 10 min and applied to air-dried slides carrying denatured metaphase chromosomes under a 22 × 22 mm coverslip. The slides were then placed in a moist chamber and incubated overnight at 37 °C. After overnight incubation, the coverslips were removed, and the slides were rinsed at 43 °C in 2 × SSC with 50% formamide twice for 15 min each, 2 × SSC for 5 min, and 1 × SSC for 5 min and then air-dried. Spectral signals were obtained by administering 8 μl of 5 μg/ml Fluorescein isothiocyanate (FITC)-conjugated antidigoxigenin antibody derived from sheep (Roche, Germany) and finally incubating in a humidity chamber at 37 °C. After a series of washes with TNT (containing 0.1 M Tris-HCl, 0.15 M NaCl, 0.05% Tween 20) at 43 °C, the slides were covered in antifade solution containing 2 μg/ml DAPI for 5 min. For each type of fish, 60 complete metaphase spreads with 48 chromosomes (20 YT, 20 TC, and 20 2nYC) were analysed under a Leica inverted CW4000 microscope and a Leica LCS SP2 confocal imaging system (Leica, Germany). Captured images were coloured and overlapped in Adobe Photoshop CS6.

### Histological observation of the hybrid gonads

At 2 years of age, 5 2nYC individuals were selected at random for histological observation of the gonadal structure via paraffin sectioning. The ovaries were fixed in Bouin’s solution, dehydrated in an increasing ethanol gradient, cleared in xylene, embedded in paraffin, sectioned using a Leica RM2016 microtome (Germany) and stained with haematoxylin and eosin. Ovary sections were examined and photographed using an Olympus microscope CX41 (Japan), and ovarian development in each sample was classified based on the previous standards for cyprinid fish [58].

## Data Availability

All the datasets used and/or analysed throughout the present study are available from the corresponding author on reasonable request.

## Abbreviations

2nYC: Diploid hybrids
4nAT: Allotetraploid hybrids
ANOVA: Analysis of variance
BH/BL: Body height to body length
BL/WL: Body length to whole length
BSB: Blunt snout bream
CC: Common carp
CPH/CPL: Caudal peduncle height to caudal peduncle length
DAPI: 4′,6-diamidino-2-phenylindole
FISH: Fluorescence in situ hybridization
GC: Grass carp
HH/BH: Head height to body height
HH/HL: Head height to head length
HL/BL: Head length to body length
HMG: High-mobility group
IE: Internal element
NTS: Non-transcribed spacer
PCR: Polymerase chain reaction
RCC: Red crucian carp
rDNA: Ribosomal DNA
rRNA: Ribosomal RNA
TC: Topmouth culter
YT: Bleeker’s yellow tail
